# A comprehensive genomic study of 390 H3F3A-mutant pediatric and adult diffuse high-grade gliomas, CNS WHO grade 4

**DOI:** 10.1007/s00401-023-02609-6

**Published:** 2023-07-31

**Authors:** Erik A. Williams, Priscilla K. Brastianos, Hiroaki Wakimoto, Amir Zolal, Mariella G. Filbin, Daniel P. Cahill, Sandro Santagata, Tareq A. Juratli

**Affiliations:** 1 Department of Pathology and Laboratory Medicine, University of Miami, Sylvester Comprehensive Cancer Center, and Jackson Memorial Hospitals, Miami, USA; 2 Foundation Medicine Inc, Cambridge, USA; 3 Department of Medicine, Massachusetts General Hospital Cancer Center, Harvard Medical School, Boston, USA; 4 Department of Neurosurgery, Laboratory of Translational Neuro-Oncology, Massachusetts General Hospital Cancer Center, Harvard Medical School, Boston, USA; 5 Department of Neurosurgery, Division of Neuro-Oncology, Faculty of Medicine and University Hospital Carl Gustav Carus, Technische Universität Dresden, 01307 Dresden, Germany; 6 Department of Pediatric Oncology, Dana-Farber Boston Children’s Cancer and Blood Disorders Center, Boston, MA USA; 7 Broad Institute of MIT and Harvard, Cambridge, MA USA; 8 Department of Pathology, Brigham and Women’s Hospital, Harvard Medical School, Boston, USA; 9 Department of Systems Biology, Harvard Medical School, Boston, USA; 10 Laboratory of Systems Pharmacology, Harvard Medical School, Boston, USA; 11 National Center for Tumor Diseases (NCT), Partner Site Dresden, Dresden, Germany

**Keywords:** Molecular pathology, Neuropathology, Neuroepithelial neoplasms, Glioma, Diffuse intrinsic pontine glioma, Brain neoplasms, Supratentorial neoplasms, Infratentorial neoplasms, Brain stem neoplasms, Genomics, Genetics, Central nervous system neoplasms, Astrocytoma, Glioblastoma, H3F3A, FGFR1, CDKN2A, PDGFRA

## Abstract

Malignant brain tumors, known as H3K27-altered diffuse midline glioma (DMG) and H3G34-mutant diffuse hemispheric glioma (DHG), can affect individuals of all ages and are classified as CNS WHO grade 4. We comprehensively characterized 390 H3F3A-mutant diffuse gliomas (201 females, 189 males) arising in pediatric patients (under 20 years old) and adults (20 years and older) evaluated by the CGP program at Foundation Medicine between 2013 and 2020. We assessed information from pathology reports, histopathology review, and clinical data. The cohort included 304 H3K27M-mutant DMG (156 females, 148 males) and 86 H3G34-mutant DHG (45 females, 41 males). Median patient age was 20 years (1–74 years). The frequency of H3K27M-mutant DMG was similar in both pediatric and adult patients in our cohort—48.6% of the patients were over 20 years old, 31.5% over 30, and 18% over 40 at initial diagnosis. *FGFR1* hotspot point mutations (N546K and K656E) were exclusively identified in H3K27M-mutant DMG tumors (64/304, 21%; p = 0.0001); these tend to occur in older patients (median age: 32.5 years) and mainly arose in the diencephalon. H3K27M-mutant DMG had higher rates of mutations in *NF1* (31.0 vs 8.1%; p = 0.0001) and *PIK3CA/PIK3R1* (27.9% vs 15.1%; p = 0.016) compared to H3G34-mutant DHG. However, H3G34-mutant DHG had higher rates of targetable alterations in cell-cycle pathway genes (*CDK4* and *CDK6* amplification; *CDKN2A*/*B* deletion) (27.0 vs 9.0%). Potentially targetable *PDGFRA* alterations were identified in ~ 20% of both H3G34-mutant DHG and H3K27M-mutant DMG. Overall, in the present study H3K27M-mutant DMG occurred at similar rates in both adult and patient patients. Through our analysis, we were able to identify molecular features characteristic of DMG and DHG. By identifying the recurrent co-mutations including actionable *FGFR1* point mutations found in nearly one-third of H3K27M-mutant DMG in young adults, our findings can inform clinical translational studies, patient diagnosis, and clinical trial design.

**Supplementary Information:**

The online version contains supplementary material available at 10.1007/s00401-023-02609-6.

## Introduction

Diffuse high-grade gliomas (HGG) are aggressive brain tumors that affect people of all ages and can occur in different regions of the central nervous system (CNS). In 2021, the World Health Organization (WHO) updated its classification of adult type and pediatric-type diffuse gliomas [20]. Adult-type diffuse gliomas are classified into three groups (i) Astrocytoma, IDH-mutant, (ii) Oligodendroglioma, IDH-mutant and 1p/19q-codeleted, and (iii) Glioblastoma, IDH-wildtype. Pediatric-type diffuse high grade glioma are classified into four groups (i) Diffuse midline glioma (DMG) H3 K27-altered, (ii) Diffuse hemispheric glioma (DHG) H3 G34-mutant, (iii) Diffuse pediatric-type HGG, H3-wildtype and IDH-wildtype, and (iv) Infant-type hemispheric glioma, typically with receptor tyrosine kinase (RTK) fusions [20].

Diffuse midline gliomas, H3K27-altered, is a highly aggressive tumor which arises in the midline in children, often in the pons [16, 25, 29]. DMG in adolescents and adults more often develop in the spinal cord or unilaterally in the thalamus [16, 25, 29]. These tumors often harbor mutations in the *H3F3A* gene which results in the substitution of the amino acid lysine with methionine at position 28 of the H3 histone protein (referred to as H3 K27M). This mutation inhibits the function of EZH2 subunit of PRC2 and promotes oncogenesis [15, 18]. In a subset of DMG, histone mutations are not observed but overexpression of EZHIP or *EGFR* mutation mimic the oncogenic properties of H3 K27M [1, 11, 30]. Mutations in *TP53*, *ATRX*, and components of the PI3K pathway have been reported to co-occur in H3K27-altered DMG*.*

Diffuse hemispheric glioma, H3G34-mutant (DHG) is a malignant tumor that typically arises in the cerebral hemispheres of teenagers and young adults. It is defined by mutations in the H3F3A gene that result in the substitution of glycine with arginine (G34R) or in about 5% of cases valine (G34V) of the H3 histone protein. These tumors display typical glioblastoma-like histomorphology or features resembling CNS embryonal tumors. Over 90% of DHG have co-occurring mutations in *TP53* and *ATRX* [29, 32].

In prior analyses, our group, along with others, conducted preliminary assessments focusing on pediatric patients. These initial assessments provided valuable insights into co-occurring mutations in H3K27-altered and H3G34-mutant pediatric-type diffuse HGG [5, 9, 12, 13, 22, 29, 32]. However, to gain a more comprehensive understanding, it is crucial to more deeply characterize the patterns of co-mutation in both adult and pediatric populations using large cohorts of these tumor types. To address this gap in information, we conducted a cross-sectional study where we present comprehensive genomic profiling of the largest set of H3K27-altered DMG (n = 304) and H3G34-mutant DHG (n = 86) that have been reported to date. Our findings significantly advance our understanding of the molecular profiles of DMG and DHG, providing critical insights that may have implications for the development of genomically-guided precision medicine trials.

## Methods

### Tumor samples and molecular genetic analysis

Samples from 402 *H3F3A*-mutant tumors that were analyzed between 2013 and 2020 as part of clinical care for patients using comprehensive genomic profiling (CGP) in a Clinical Laboratory Improvement Amendments-certified, College of American Pathologists-accredited laboratory (Foundation Medicine, Cambridge, MA). The samples were profiled from a wide range of referring institutions in the US. The large size of the cohort and the large panel of genes make our study powered to examine multiple clinical associations and gene–gene pathway interactions that define specific genomic tumor subgroups.

Within this cohort of 402 *H3F3A*-mutant gliomas, 59 (~ 15%) were previously included in a study characterizing genomic alterations in low and high grade pediatric gliomas [12]. We carefully considered the STROBE criteria to ensure that our study was designed and reported with the highest level of quality and transparency. Moreover, approval for this study, including a waiver of informed consent and a HIPAA waiver of authorization, was obtained from the Western Institutional Review Board (Protocol No. 20152817). The pathologic diagnosis of each case was first made in the referring center and was then confirmed in our facility (Foundation Medicine, Cambridge, MA) on routine hematoxylin and eosin stained slides. All samples that contained a minimum of 20% tumor nuclear nuclei were forwarded for DNA and/or RNA extraction. The analysis was performed using a next-generation sequencing panel that covers more than 450 genes. Technical descriptions and validation of the genomic profiling assays used to analyze these samples in the course of clinical care have been published previously [8, 10]. In brief, ≥ 50 ng DNA was extracted from 40 µm scrolls from formalin-fixed, paraffin-embedded (FFPE) tissue blocks of tumor. The samples were assayed by adaptor ligation hybrid capture, performed for all coding exons of 236 (v1), 315 (v2), or 405 (v3) cancer-related genes plus select introns from 19 (v1), 28 (v2), or 31 (v3) genes frequently rearranged in cancer [8, 10]. For those samples for which RNA was available, targeted RNA-seq was performed for rearrangement analysis in 265 genes. RNA sequences were analyzed for the presence of rearrangements only. Sequencing of captured libraries was performed using an Illumina technology to a mean exon coverage depth of 593 × , and resultant sequences were analyzed for base substitutions, insertions, deletions, copy number alterations (focal amplifications and homozygous deletions), and select gene fusions, as previously described [8, 10]. Clinically relevant genomic alterations (CRGA) were defined as alterations that are targetable by anticancer drugs currently available on the market or in registered clinical trials. Germline variants documented in the dbSNP database (dbSNP142; http://www.ncbi.nlm.nih.gov/SNP/), with two or more counts in the ExAC database (http://exac.broadinstitute.org/), or recurrent variants of unknown significance that were predicted by an internally developed algorithm to be germline were removed, with the exception of known driver germline events (e.g., documented hereditary *BRCA1/2* and deleterious *TP53* mutations) [33]. Known confirmed somatic alterations deposited in the Catalog of Somatic Mutations in Cancer were highlighted as biologically significant [7]. To maximize mutation-detection accuracy (sensitivity and specificity) in impure clinical specimens, the test was previously optimized and validated to detect base substitutions at a ≥ 5% mutant allele frequency (MAF), indels with a ≥ 10% MAF with ≥ 99% accuracy, and fusions occurring within baited introns/exons with > 99% sensitivity.

### Statistical analysis

The statistical association of detected somatic alterations with other factors, including age, sex, and tumor location were analyzed using the Fisher exact and Mann–Whitney-U tests. Cases with unavailable molecular or histology data were excluded from the final correlation analysis. A two-tailed p value of < 0.05 was considered to be statistically significant. Furthermore, R statistics system version 3.6.1 (https://www.r-project.org/) together with the UpSetR [3] library was used to construct the diagrams of set intersections used in this paper.

Moreover, by carefully considering the STROBE criteria in the design and reporting of our study, we ensure that our research is of the highest quality and transparency, to enable other researchers to accurately interpret and build upon our findings.

## Results

### Cohort description

We collected data from 402 *H3F3A*-mutant gliomas that were profiled in the comprehensive genomic profiling (CGP) program at Foundation Medicine between 2013 and 2020. Among these, we identified six cases of *H3F3A*-mutant ganglioglioma and six cases that had both *H3F3A* and *IDH* mutations (4 *IDH1* R132H and 2 *IDH2* R172C). These twelve cases were excluded from subsequent analyses.

#### Demographics and clinical characteristics of patients with H3F3A mutations

**Fig. 1:**
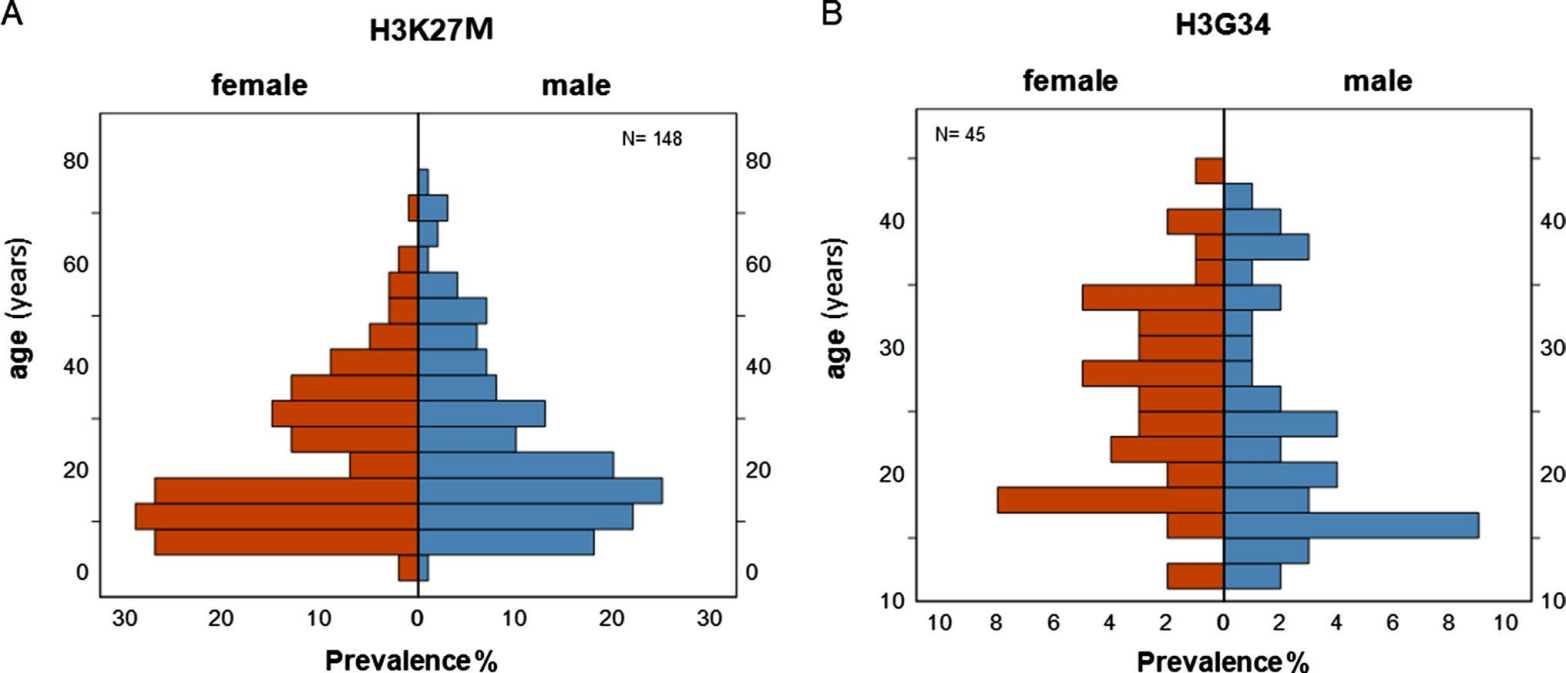
Age and sex distribution of 390 patients with *H3F3A*-mutant gliomas including **a** 304 with H3K27M-mutant Diffuse Midline Glioma and **b** 86 with H3G34-mutant Diffuse Hemispheric Glioma

From the 390 patients with *H3F3A*-mutant diffuse gliomas, 201 were female and 189 male. The median age was 20 years, ranging from 1 to 74 years. 304 of these patients (77.9%; 156 female, 148 male) had H3K27M-mutant DMG. 86 had H3G34*-*mutant DHG (45 female, 41 male). The median age of H3K27M-mutant DMG (19 years) differed significantly from patients with H3G34-mutant DHG (23 years; p = 0.021). 47.9% of patients were pediatric (younger than 20 years old) (n = 187/390). In our cohort, a higher proportion of pediatric patients had H3K27M versus H3G34-mutant gliomas (n = 156/304, 51.3%, v. n = 31/86, 36%; p = 0.014). We found that female patients with H3K27M-mutant gliomas were younger at the time of initial diagnosis than males (median age: 18 years vs 20 years; p = 0.14) whereas males with H3G34-mutant DHG were younger than females at the time of initial diagnosis (20 v. 25 years, p = 0.084) (Fig. 1). All H3G34-mutant DHG arose within the hemispheres of the brain. However, eight of 304 H3K27M-mutant gliomas developed within the spinal cord (2.6%). This analysis provides insights into both the demographics as well as clinical characteristics of patients with *H3F3A*-mutant diffuse gliomas. It also reveals important differences between patients with H3K27M-mutant DMG and H3G34-mutant DHG.

### Genetic correlations

In this analysis of 390 *H3F3A*-mutant gliomas, we found that H3K27M and H3G34 mutations were mutually exclusive. Of the H3 G34 mutations (n = 86), nearly all were present as three different substitutions: (i) c.103G > A p.Gly35Arg (G34R in 64/86 cases, 74.5%), (ii) c.103G > C p.Gly35Arg (G34R in 11/86 cases, 12.8%), and (iii) c.104G > T p.Gly35Val (G34V in 8/86 cases, 9.3%). Two additional variants were infrequently detected: two of c.104G > A p.Gly35Glu (G34E) and one of c.103_104GG > TT p.Gly35Leu (G34L).

**Fig. 2:**
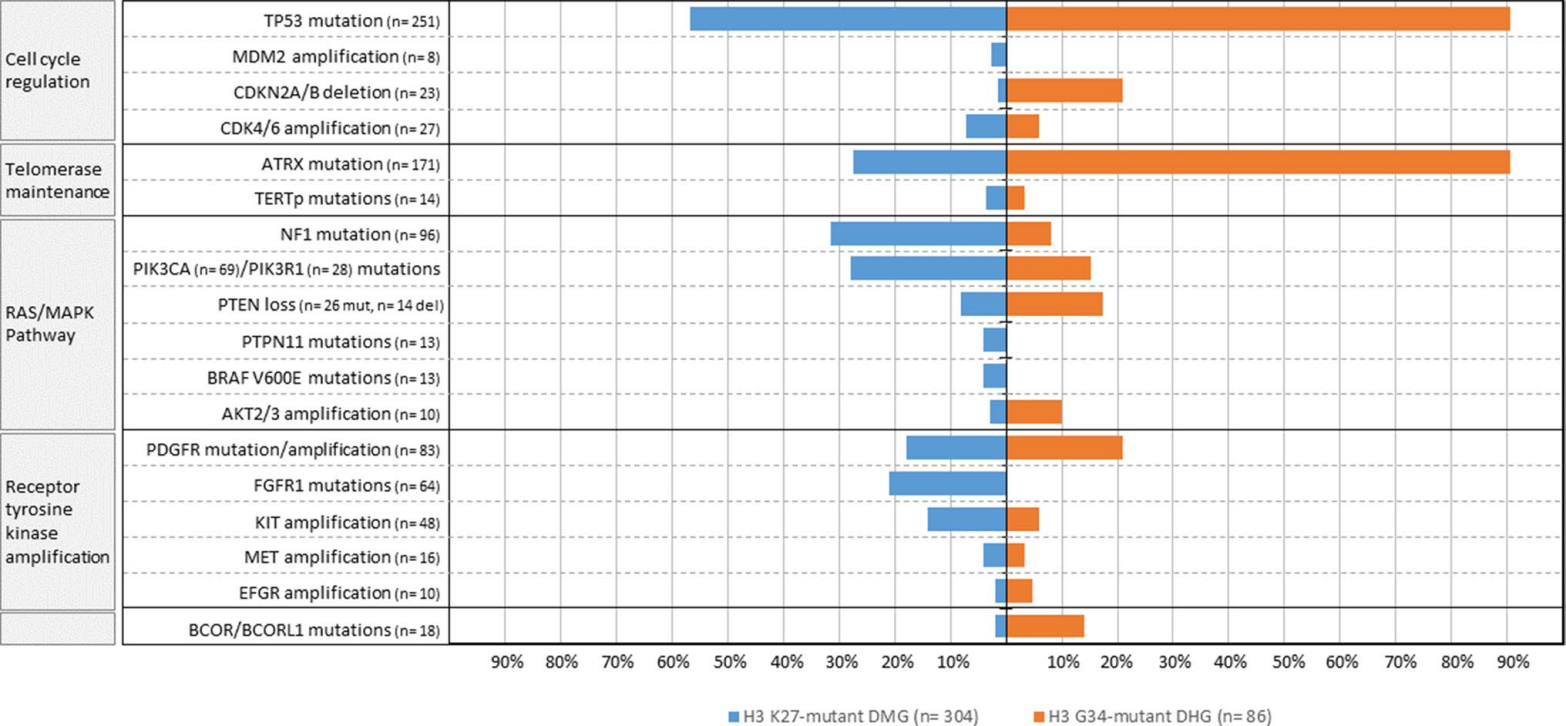
Comparison of recurrent genomic alterations between 304 patients with H3 K27M-mutant Diffuse Midline Glioma, and 86 H3 G34-altered Diffuse Hemispheric Glioma

Our analysis of this cohort revealed that the most frequent genomic alterations found in conjunction with H3 K27M and H3 G34V/R were in common oncogenes and tumor suppressors: *TP53* (n = 251, 64.3%), *ATRX* (n = 171, 43.8%), *NF1* (n = 96, 24.6%), *PIK3CA* (n = 69, 17.7%), *PIK3R1* (n = 28, 7.1%), *FGFR1 (*n = 64, 6.4%)*, PDGFRA* (n = 22, 5.6%), *PTEN* (n = 26, 6.66%), *PTPN11* (n = 13, 3.3%), *BCOR/BCORL1* (n = 18, 4.6%), *BRAF (*n = 13, 3.3%*)* and the *TERT* promoter (n = 14, 10 mutations at position -228 and four at position -250). We also observed recurrent copy number alterations which included deletions of *CDKN2A/B* (n = 23, 5.9%; 18 homozygous deletion and 5 heterozygous deletion) and *PTEN* (n = 14, 3.6%). Additionally, amplifications of *PDGFRA* (n = 51, 13%), *KIT* (n = 48, 12.3%), *CDK4/6* (n = 27, 6.9%), *MET* (n = 16, 4.1%), *EGFR* (n = 10, 2.5%), *AKT2/3* (n = 10, 2.5%), *MDM2* (n = 8, 2%), *MYCN* (n = 9, 2.3%), and *MYC* (n = 7, 1.8%) were identified*.* We also found that other known cancer-related genes were also altered at low frequency including *APC* (n = 5), *KRAS* (n = 2), *SETD2* (n = 2), *DAXX* (n = 1) and *STAG2* (n = 1) (Fig. 2).

### The molecular profile of H3K27M-mutant DMG

Our analysis revealed that *NF1* (31% v. 8.1%; p = 0.0001) and *PIK3CA/PIK3R1* (27.9% v. 15.1%; p = 0.016) were more frequently mutated in H3K27M-mutant DMG than in H3G34-mutant DHG, in this cohort (Fig. 2, Supplementary Table 1). *FGFR1* mutations were identified exclusively in H3K27M-mutant DMG (n = 64/304, 21%; p = 0.0001). *BRAF*^*V600E*^ and mutations in *PTPN11,* two genes encoding components of the RAS/MAPK pathway, were also found exclusively in H3K27M-mutant DMG (13 cases with mutation in each of these genes). *PDGFRA* amplifications were present in a slightly higher fraction of the H3K27M-mutant DMG (n = 41, 13.4%) than the H3G34-mutant DHG (n = 10, 11.6%; p = 0.72), By contrast, *PDGFRA* mutations were less frequent in H3K27M-mutant DMG (n = 14, 4.6%) compared with H3G34-mutant DHG (n = 8, 9.3%, p = 0.11). *KIT* amplifications were significantly more common in H3K27M-mutant DMG (14.1% v. 5.8%; p = 0.040).

### Age-specific mutation profiles of H3K27-mutant DMG

**Fig. 3:**
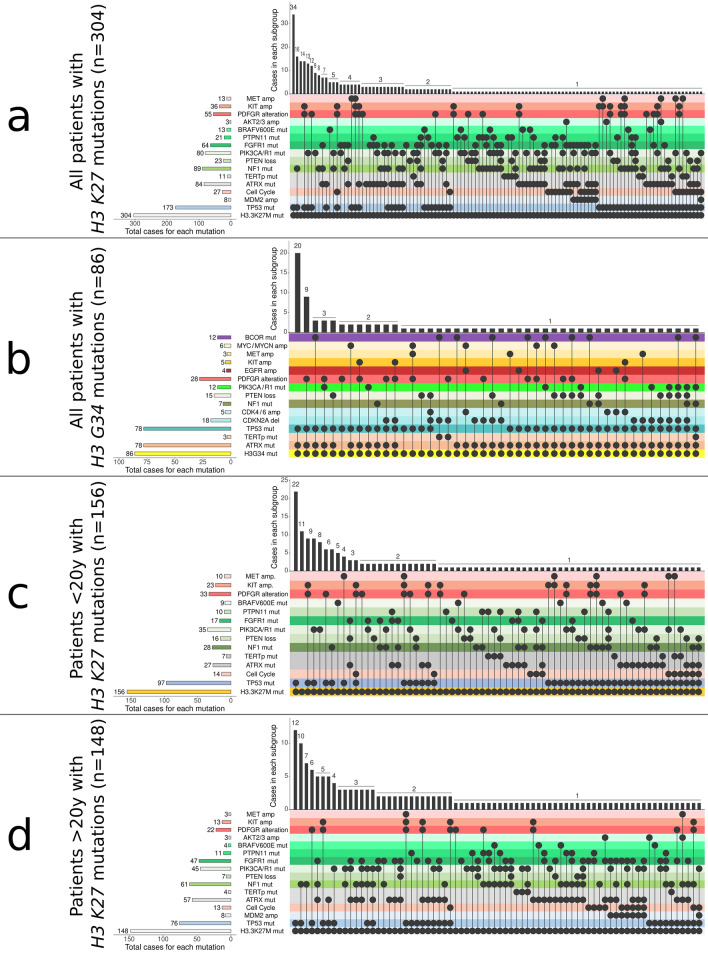
Mutation spectrum of 390 patients with H3F3A-mutant gliomas. **a** Mutation spectrum of all 304 patients with H3K27M DMG. **b** Mutation spectrum of 156 patients < 20 years with H3K27M DMG. **c** Mutation profile of 86 patients with H3G34-mutant DHG. **d** Mutation spectrum of 148 patients ≥ 20 years with H3K27M DMG. Cell cycle category includes CDK4/6 amplification and CDKN2A/B deletions

Next, we compared the mutational profiles of H3K27-mutant DMG between pediatric patients (n = 156) with those of patients 20 years old or older (n = 148) to investigate whether there are age-specific genomic alterations in pediatric and adult patients in our cohort. We found that mutations in *NF1* and *ATRX* occurred more commonly in H3K27-mutant DMG in patients older than 20 years old in comparison with tumors arising in pediatric patients (*NF1*: 41.2% v. 18%; *ATRX*: 38.5% v. 17.3%, both with p = 0.0001). In addition, we observed that mutations in *FGFR1* were more common in tumors from adult patients (31.7% v. 11%, p = 0.0001), while tumors arising in pediatric patients more often had *PTEN* loss (11.5% v. 4.7%; p = 0.036) (Table 1, Fig. 3b, d).

**Table 1:** Comparison of the frequency of recurrent genomic alterations in patients with H3K27M-mutant DMG by age of first diagnosis (< 20 years vs. ≥ 20 years)

	Genomic alteration	Patients < 20 years old with H3K27-mutant DMG (n = 156)	Patients ≥ 20 years old with H3K27-mutant DMG (n = 148)	p-value
Cell cycle regulation	*TP53 *mutation (n = 173)	97 (62.2%)	76 (51.4%)	n.s
	*MDM2* amplification (n = 8)	0%	8 (5.4%)	**0.0029**
	*CDKN2A/B* deletion (n = 5)	4 (2.6%)	1 (0.7%)	n.s
	*CDK4/6 *amplification (n = 22)	10 (6.4%)	12 (8.1%)	n.s
Telomerase maintenance	*ATRX* mutation (n = 84)	27 (17.3%)	57 (38.5%)	** < 0.001**
	*TERTp* mutation (n = 11)	7 (4.5%)	4 (2.7%)	n.s
RAS/MAPK Pathway	*NF1* mutation (n = 89)	28 (17.9%)	61 (41.2%)	**0.0001**
	*PIK3CA/PIK3R1* mutation (n = 80)	35 (22.4%)	45 (30.4%)	n.s
	*FGFR1* mutation (n = 64)	17 (10.9%)	47 (31.8%)	**0.0001**
	*PTEN* loss (n = 25) (15 mutation, 10 deletion)	18 (11.5%)	7 (4.7%)	**0.036**
	*PTPN11* mutation (n = 21)	10 (6.4%)	11(7.4%)	n.s
	*BRAF*^*V600E*^ mutation (n = 13)	9 (5.8%)	4 (2.7%)	n.s
	*AKT2/3* amplification (n = 7)	4 (2.6%)	3 (2%)	n.s
Receptor tyrosine kinase amplification	*PDGFRA* mutation (n = 14)	8 (5.1%)	6 (4.1%)	n.s
	*PDGFRA* amplification (n = 41)	25 (16%)	16 (10.8%)	n.s
	*KIT* amplification (n = 36)	23 (14.7%)	13 (8.8%)	n.s
	*MET* amplification (n = 13)	10 (6.4%)	3 (2%)	n.s
	*EGFR* amplification (n = 6)	3 (2%)	3 (2%)	n.s
	*BCOR/BCORL1* mutation (n = 6)	3 (2%)	3 (2%)	n.s

### H3K27M-mutant DMG with co-occurring *FGFR1* mutations

**Fig. 4:**
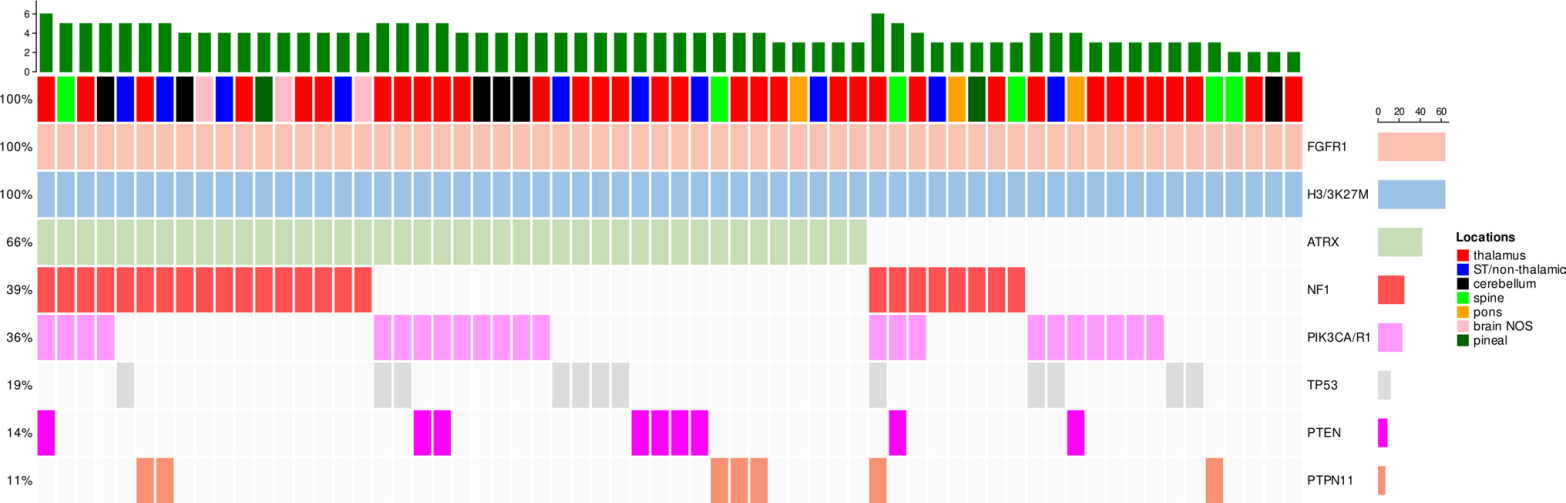
Mutation spectrum in H3K27M-mutant DMG with co-occurring mutations in FGFR1. *Loc*: location, *ST* supratentorial

The cohort included 64 H3K27M-mutant gliomas (35 females and 29 males) that had point mutations in *FGFR1*. 72% of these were N546K mutations and 28% were K656E, both occurring in the intracellular protein kinase domain of *FGFR1* (Fig. 4). 71.9% of the *FGFR1* mutations (n = 46/64) occurred in patients ≥ 20 years old at the time of initial diagnosis. In this cohort, there was a large difference in the median age of patients harboring both H3K27M and *FGFR1* mutations (32.5 years, range 10 to 74 years) when compared with the H3K27M-mutant*/FGFR1*-wildtype group (16 years, p = 0.001) or the median age of patients in the H3K27M-mutant group (19 years). Interestingly, in this cohort, we identified *TP53* mutations in only 12 of the H3K27M*/FGFR1*-mutant group (18.8%) compared with 161 of the H3K27M-mutant*/FGFR1*-wildtype group (67%, p = 0.0001). By contrast, *ATRX*-mutations were more common in the H3K27M*/FGFR1*-mutant group (n = 42, 65.5%) compared with H3K27-mutant/*FGFR1*-wildtype group (n = 43, 18%; p = 0.0001).

A distinctive clinical feature of H3K27M/*FGFR1*-mutant DMG was their predominant location in the midline of the brain but outside of the brainstem, as determined from review of radiology reports. Three H3K27M/*FGFR1-*mutant gliomas (4.6%) developed in the brainstem, while many more arose in the thalamus (n = 34, 53%). In addition these tumors arose at other sites outside of the brainstem including the supratentorial non-thalamic locations (n = 10, 15.6%), the spinal cord (n = 6, 9.3%), the cerebellum (n = 6, 9.3%) and the pineal gland (n = 2, 3%) (Fig. 4). Tumor location was unavailable for 3 cases.

### The molecular profile of H3G34-mutant DHG

In this cohort, we found that certain genomic alterations occurred frequently in H3G34-mutant DHG—*TP53* mutations were commonly detected (78 of 86 cases; 90.7%) compared with H3K27M-mutant group (n = 173 of 304, 56.9%, p = 0.0001) (Figs. 2, 3c). Similarly, *ATRX* and H3G34 mutations co-occurred frequently (n = 78/86, 90.7%) compared with H3K27M mutants (n = 84/304, 27.6%, p = 0.0001) (Fig. 2 and Supplementary Table 1). In addition, both mutations (n = 11) or deletions (n = 4) in *PTEN* were more common in H3G34-mutant gliomas than in H3K27M-mutant gliomas (17.4% v. 8.2%; p = 0.0024).

Although not frequent, *CDKN2A/B* deletions (either one or two copy deletion) were significantly more common in H3G34-mutant DHG (n = 18/86, 21%) than in H3K27-mutant DMG (n = 5/304, 1.6%, p = 0.0001; Fig. 2 and Supplementary Table 1). Of the 23 cases with deletion of *CDKN2A*, 5 had heterozygous deletions, and all of those were present in the H3G34-mutant gliomas (Fig. 2 and Supplementary Table 1). Other alterations such as *PDGFRA* amplification (n = 10), *TERT*p mutations (n = 3), amplification of *MYC*/*MYCN* (n = 6), *MET* (n = 3), and *EGFR* (n = 3) did not differ significantly between H3G34-mutant DHG and H3K27M*-*mutant DMG.

## Discussion

Our cross-sectional study analyzed the demographics, clinical characteristics, and genetic correlations of 390 cases of *H3F3A*-mutant pediatric-type HGG, a rare type of brain tumors that can affect both children and adults. The study yielded important insights into these aggressive tumors, both confirming previously published observation and revealing new findings. Notably, while H3K27M-mutant DMG is rare when compared with *IDH*-wildtype glioblastoma in adult patients, our cohort showed that H3K27M-mutant DMG are as common in adult patients as they are in pediatric patients, despite their designation as ‘pediatric-type’ gliomas. Female patients with H3K27M-mutant DMG were approximately two years younger at initial diagnosis compared with males, whereas male patients developed H3G34-mutant DHG five years earlier than female patients.

The majority of patients in our study (78%) had H3K27M-mutant DMG, and the median age of this group was significantly different from those with H3G34-mutant DHG. A higher proportion of patients < 20 years had H3K27M-mutant compared with H3G34-mutant tumors. H3K27M and H3G34 mutations were mutually exclusive. The most common genomic alterations co-occurring with H3 K27M and H3 G34V/R were in common tumor suppressors genes: *TP53*, *ATRX,* and *NF1*. In H3G34-mutant DHG tumors, we found *TP53* and *ATRX* mutations in more than 90% of cases, consistent with other reports in the literature [14, 29, 32]. Interestingly, while *IDH* mutations have been reported to be absent from mutant H3.3. tumors [16, 29, 32], six cases had simultaneous *H3F3A* and *IDH* mutations (4 IDH1 R132H and 2 IDH2 R172C). These tumors and *H3F3A*-mutant gangliogliomas were excluded from our analysis as they may represent distinct entities and pathophysiology.

Moreover, we identified several canonical gene alterations in cancer-associated pathways with subtype-specific enrichment in DHG and DMG. Importantly, we detected recurrent *FGFR1* mutations (21%), in addition to less frequent *PTPN11* and *BRAF*^*V600E*^ mutations (4.3% each) that occurred exclusively in H3K27M-mutant DMG. *FGFR1* is a growth factor receptor that represents the second most commonly altered gene in pediatric-type low grade gliomas, such as rosette-forming glioneural tumors, and dysembryoplastic neuroepithelial tumors [26, 35]. Fontebasso et al. first reported the association between activating mutations in *FGFR1* and H3K27M alterations in 4 of 39 DMG, three of which were located in the thalamus [6]. Of the 64 *H3K27M/FGFR1* double mutant DMG from our cohort, we also observed frequent origin in the thalamus (53%), while only three cases developed in the brainstem (pons). Liu et al. reported single-cell multi-omic and spatial transcriptomic results from 50 H3K27M-mutant DMG encompassing a broad range of age groups and anatomical locations, including three patients harboring *FGFR1* mutations with tumor located in the thalamus [19].

Of the nine cases with *FGFR1* mutations identified in a series of 83 H3K27M-mutant DMG [28], Schueller et al. noted a reciprocal association between *TP53* and *FGFR1* mutations, a finding that we also observe in our study and others have confirmed elsewhere [36]. We add that *ATRX* mutations are significantly enriched in dual H3K27M/*FGFR1*-mutant DMG in comparison with their H3K27M*-*mutant*/FGFR1*-wildtype counterparts. Our study is the first to demonstrate an association between mutant *FGFR1* in DMG and a higher age of first diagnosis (median age 32.5 years), as well as a wide distribution of tumors developing across the diencephalon, including areas of the thalamus, pineal gland, and supratentorial regions that are adjacent to the thalamus. This pattern of tumor may be explained by the role of *FGFR1* in neural development, in particular of the diencephalon area [24, 27]. Nevertheless, several *FGFR1*-mutant cases were located in the spinal cord or cerebellum. Notably, Schueller et al. reported that patients with H3K27M/*FGFR1*-mutant DMG had a better prognosis compared with patients harboring H3K27M/*TP53*-mutant DMG [28]. This finding has been confirmed in a recent study which aggregated clinical and genomic information from 30 studies including 669 H3-DMGs, underscoring the prognostic value of *FGFR1* mutations in DMG [36]. The high frequency of *FGFR1* point mutations in H3K27M-mutant DMG (~ 21% of all patients as revealed in our study), indicates the importance of designing clinical trials to investigate the effects of targeted inhibitors in this subset of patients, as FGFR-targeting therapies have already been introduced in several non-CNS malignancies [31, 34]. A single-arm, multicenter, phase II study of infigratinib (BGJ398) showed durable responses observed in a small number of patients with HGGs with *FGFR1* point mutations, including one patient with an H3K27M-mutant/FGFR1 K656E-mutant DMG who had a partial durable response and progression free survival of 21.9 months [4, 17]. While ~ 10% of pediatric patients with H3K27M-mutant DMG have *FGFR1*-mutations, nearly one third of H3K27M DMG in young adults (≥ 20 years old) harbor *FGFR1* point mutations; HGG arising in a thalamic location (or diencephalic areas in a wider sense) in this population should prompt screening for H3K27M and for actionable *FGFR1* mutations.

Furthermore, our analysis revealed that *NF1*, *PIK3R1* and *PIK3CA* are commonly mutated genes in *H3F3A*-mutant gliomas (24.6%, 17.7%, and 7.7%, respectively). Notably, they are more frequently mutated in H3K27M-mutant DMG than in H3G34-altered DHG. Subsequently, PIK3CA inhibitors may warrant investigation for the treatment of these brain tumors. A trial of BKM120 (buparlisib), a small-molecule inhibitor of the PI3K signaling pathway, demonstrated inhibition of the PI3K/AKT/mTOR signaling, which is often overactive in tumor cells [37]. Isoform-specific inhibitors of PI3K have been shown to increase efficacy and reduce side effects [23]. One major barrier to the use of these inhibitors for treating brain tumors is blood–brain barrier penetration [2]. Developing inhibitors with improved blood–brain barrier penetration remains a major focus in this field [2].

Other frequently detected co-alterations were *PDGFRA* amplification and *CDKN2A/B* deletion. *PDGFRA* amplifications were slightly more common in H3K27M-mutant DMG than in H3G34-mutant DHG. However, *PDGFRA* mutations were less common in H3K27M-mutant DMG compared to H3G34-mutant DHG. Additionally, genomic profiling revealed that *CDKN2A/B* deletions occur in 21% of H3G34-mutant DHG while these deletions were uncommon in H3K27-mutant DMG in our cohort. In a recent study, Lucas et al. performed genomic characterization of 10 DHG which revealed seven alterations in *PDGFRA* and three alterations affecting the CDK4/6-cyclin D-p16INK4a-Rb cell cycle pathway [21]. The authors discussed the possibility of targeting DHG with mutant *PDGFRA* and/or activated *CDK4/6* using small molecule kinase inhibitors. In our comprehensive study, we show that potentially targetable *PDGFRA* alterations are present in one-fifth of H3G34-mutant DHG and H3K27M-mutant DMG. Similarly, we show that targetable alterations in cell-cycle pathway components (e.g., *CDK4/6* amplifications, *CDKN2A*/*B* deletions) are more common in H3G34-mutant DHG (27%) than H3K27M*-*mutant DMG (9%). This finding suggests that patients with H3G34-altered DHG may be more likely to benefit from targeted treatment with CDK4/6 inhibitors.

While our study provides valuable insights into the molecular and genetic features of pediatric-type high grade gliomas, it is important to acknowledge several limitations. The most significant limitation is the incomplete availability of clinical information for patients whose tumors were analyzed as part of the comprehensive genomic profiling (CGP) program. The lack of survival and treatment data underscores a key need for additional studies of large patient cohorts that are designed to collect both molecular tumor data and patient outcome information including detailed data about tumor progression and response to therapy.

Furthermore, it is important to acknowledge that our cross-sectional study is not based on a population sample. This feature introduces the possibility of selection bias because certain cases may have been referred for genomic profiling more often than others. Attempts at generalizing these findings to a broad population of patients should consider this limitation. Additionally, our analysis does not include cases of *H3.1* mutant gliomas, as the NGS panel used for tumor analysis does not cover mutations in *H3.1*, and this limitation should be considered when interpreting the demographics observed. Moreover, it is important to note that the likelihood of biopsy from pontine tumors is generally lower compared to thalamic tumors, and tissue adequacy from attempted pontine biopsies is often limited. A discrepancy in biopsy rate and perhaps the amount of sample available for ancillary genetic testing, may also introduce a bias that could impact the results and the interpretation of the findings. Future research can be planned to address these limitations and provide a deeper understanding of the clinical and molecular features of pediatric-type high grade gliomas.

Overall, our study expands our understanding of the tumor-specific molecular features of pediatric-type high grade gliomas that arise in both children and adults. These findings may help guide basic and translational research that is aimed at better understanding the biological pathways supporting DHG and DMG, new strategies for diagnosing and treating patients, and the design of genomically-guided clinical trials.

## Supplementary Information

Below is the link to the electronic supplementary material.

Supplementary file1 (DOCX 15 KB)

## Data Availability

The supplementary information includes all data.
