# Supplementary material for: A comprehensive genomic study of 390 H3F3A-mutant pediatric and adult diffuse high-grade gliomas, CNS WHO grade 4
Source: Acta Neuropathol. 2023 Jul 31;146(3):515–25. doi: 10.1007/s00401-023-02609-6 (PMC10412483; doi:10.1007/s00401-023-02609-6)
Supplement: Supplementary file 1 — Supplementary file1 (DOCX 15 KB) [file 401_2023_2609_MOESM1_ESM.docx]

| **All patients** | **Genomic alteration** | **H3 K27-mutant DMG (n = 304)** | **H3 G34-mutant DHG (n = 86)** | **p-value** |
| --- | --- | --- | --- | --- |
| ***Cell cycle regulation*** | ***TP53* mutation (n = 251)** | 173 (56.9%) | 78 (90.7%) | **0.0001** |
|  | ***MDM2 amplification (n = 8)*** | 8 (2.6%) | 0 | n.s. |
|  | ***CDKN2A/B* deletion (n = 23)** | 5 (1.6%) | 18 (20.9%) | **0.0001** |
|  | ***CDK4/6 amplification (n = 27)*** | 22 (7.2%) | 5 (5.8%) | n.s. |
| ***Telomerase maintenance*** | ***ATRX* mutation (n = 171)** | 84 (27.6%) | 78 (90.7%) | **0.0001** |
|  | ***TERTp* mutations (n = 14)** | 11 (3.6%) | 3 (3.5%) | n.s. |
| ***RAS/MAPK Pathway*** | ***NF1* mutation (n = 96)** | 89 (29.3%) | 7 (8.1%) | **0.0001** |
|  | ***PIK3CA*  mutations (n = 69)** | 59 (19.4%) | 10 (11.6%) | n.s |
|  | ***PIK3R1* mutations (n = 28)** | 25 (8.2%) | 3 (3.4%) | **n.s.** |
|  | ***FGFR1* mutations (n = 64)** | 64 (21%) | 0 | **0.0001** |
|  | ***PTEN* loss (n = 26 mutations, 14 deletions)** | 15/10 (8.2%) | 11/4 (17.4%) | **0.0024** |
|  | ***PTPN11* mutations (n = 13)** | 13 (4.3%) | 0 | n.s. |
|  | ***BRAF V600E* mutations (n = 13)** | 13 (4.2%) | 0 | n.s. |
|  | ***AKT2/3* amplification (n = 10)** | 9 (3%) | 1.2% | n.s. |
| ***Receptor tyrosine kinase*** | ***PDGFRA* mutation (n= 22)** | 14 (4.6%) | 8 (9.3%) | n.s. |
|  | ***PDGFRA* amplification (n =51)** | 41 (13.5%) | 10 (11.6%) | n.s. |
|  | ***KIT* amplification (n = 48)** | 43 (14.1%) | 5 (5.8%) | **0.040** |
|  | ***MET* amplification (n =16)** | 13 (4.3%) | 3 (3.5%) | n.s. |
|  | ***EGFR* amplification (n = 10)** | 6 (2%) | 4 (4.7%) | n.s. |
| ***Epigenetic regulation*** | ***BCOR/BCORL1* mutations (n = 18)** | 6 (2%) | 12 (14%) | **0.0001** |

**Supplementary Table 1:** Comparison of recurrent genomic alterations between 304 patients with *H3K27M*-mutant DMG and 86 *H3G34*-mutant DHG WHO grade 4.
